# Trauma Chronicity and the Long-Term Needs of Childhood Sexual Trauma Survivors

**DOI:** 10.3390/sexes3030028

**Published:** 2022-07-17

**Authors:** Ashley C. Schuyler, Joseph A. Catania

**Affiliations:** Hallie E. Ford Center for Healthy Children and Families, School of Social and Behavioral Health Sciences, College of Public Health and Human Sciences, Oregon State University, Corvallis, OR 97331, USA

**Keywords:** childhood sexual trauma, trauma duration, long-term health outcomes, help-seeking

## Abstract

Research has linked childhood sexual trauma (CST) with adverse adult outcomes (AAOs) that span physical, psychological, and social domains of functioning. Differences in conceptualizing and measuring CST, however, have inhibited the examination of trauma-related variables hypothesized to impact adult outcomes. We used National Sexual Health Survey (NSHS; 1995–1996) data to examine trauma chronicity (i.e., duration) and AAOs (domains: physical and mental health, close relationships, and achievement). The NSHS (*N* = 6537, 18–70 years) assessed duration using perpetrator-specific CST reports. Adjusting for background characteristics, we examined CST duration in relation to AAOs and CST-related help-seeking. Approximately 8% of participants reported CST. Chronic (vs. single-exposure) CST survivors were at substantially higher risk of experiencing AAOs [i.e., mean AAOs and specific AAOs (e.g., physical and emotional health problems, divorce/separation, and poverty)]. CST had direct effects on sexual dysfunction and satisfaction, and on relationship stressors which may impact sexual relationship quality. Although 62% of CST survivors did not seek help, those with more chronic CST histories reported a higher prevalence of trauma-related help-seeking. Our work underscores the importance of examining CST chronicity in relation to long-term developmental outcomes. Chronicity assessment may be an important screening tool in the therapeutic context and in broader community screening efforts.

## 1. Introduction

Childhood sexual trauma (CST; i.e., before age 18) is associated with a wide range of adult adverse outcomes (AAOs) across physical and mental health, social, and life-achievement domains [1–6]. Accordingly, CST is linked with increased healthcare utilization and considerable economic burden [7–9]. Mitigating the long-term health impact of CST requires the ability to deliver interventions to those most likely to experience AAOs. To accomplish this task, we need to identify and prioritize sub-populations with CST histories at risk for developing AAOs. Few studies have (a) examined trauma-related AAOs across a wide range of domains, (b) identified priority populations at high risk for AAOs, or (c) investigated trauma-related help-seeking. To our knowledge, no U.S. studies have examined all three issues within the same investigation.

### 1.1. CST: Impact on a Range of Life Domains

Trauma is an extremely adverse experience which is psychologically and physically overwhelming, has a higher degree of severity compared to other types of stressors, and, therefore, may substantially elevate the risk of long-term negative health outcomes [10–16]. CST is a complex trauma involving physical, emotional, and sexual components [12,17,18]. It may include sexual behaviors which are painful and fear-arousing, such that the sexual experience itself encompasses both physical and psychological trauma [12,17,19–22]. Moreover, CST may co-occur with other non-sexual forms of psychological and physical abuse [23–27].

From a sexual health perspective, a broader consideration of CST-related AAOs allows for an examination of the direct impact of CST on people’s sexual lives as well as indirect effects through effects on other types of relationship stressors (e.g., health problems, work stress). Prior research supports linkages between CST history and adult sexual health that may vary in relation to trauma severity and co-occurring mental health problems [28–31]. There is evidence to suggest CST might impede sexual functioning by both a) altering physiological, cognitive, and affective sexual response processes [31,32], and b) exacerbating other life stressors that may facilitate sexual difficulties [6,33,34]. The current study explores linkages between CST and sexual health-related AAOs across multiple life domains.

Browning and Laumann (1997) provided evidence to support their claim that developmental processes may link CST to sexual health outcomes. Although they argued that a developmental perspective is an alternative to trauma theory explanations of CST outcomes, as we discuss below, trauma theory and developmental perspectives on long-term CST outcomes are not inconsistent. Because CST may involve traumatic experiences at multiple levels, it may have broad-reaching effects which disrupt core developmental competencies during childhood and adolescence. These developmental challenges, in turn, produce a wide range of AAOs (i.e., physical and mental health, social relationships, and achievement domains) [2,4–6,12,18,25,35–37]. We examined the impact of CST across four major life domains. Unfortunately, only a small subset of CST studies has examined multiple domains of AAOs, and those have limited generalizability within the U.S. (e.g., non-U.S. samples [8] or nonrepresentative samples [38]).

### 1.2. Trauma Duration and AAOs

Consistent with traumatic stress theory, clinical observations and empirical studies support the following observations: (a) temporal components of stressors are key factors in determining stress outcomes [10,13,16,39–41]; (b) chronic traumas produce more severe and long-term adverse social-psychological and physical outcomes [10,12,15,40]; and (c) single event traumas, although highly stressful, have substantially less impact on long-term adverse outcomes [39,42,43]. On balance, existing evidence suggests that chronic trauma experiences may produce powerful learning contingencies (e.g., anticipatory anxiety between chronic trauma events facilitates the belief that danger is unavoidable) that facilitate developmental consequences associated with long-term adverse outcomes (e.g., development of maladaptive coping responses) [12,37,44,45]. Trauma chronicity (i.e., duration) has been underutilized as a means of stratifying CST survivors with regards to AAOs [12,40,41]. The current study will examine the relationship of CST duration to specific and cumulative AAOs.

### 1.3. CST Duration and Help-Seeking

Programs designed to mitigate sexual health problems at the population level often rely on proactive individuals seeking help for an underlying problem (e.g., seeking HIV/STI testing and treatment services, reproductive services, and prevention programs). Prior work on CST-related help-seeking has: (a) insufficiently examined trauma-related factors in relation to disclosure and help-seeking patterns, (b) lacked a life course perspective, and (c) varied in the types of help sources assessed [46,47]. The ability to target interventions to increase help-seeking (e.g., [48]) toward those with the greatest need requires, in the current context, characterizing CST survivors who are less likely to seek help. In general, CST-related help-seeking research has been limited. It is well understood that distress, particularly prolonged distress, is a primary motivator of help-seeking behavior [49–52]; thus, we would expect CST of longer duration to facilitate more help-seeking due to prolonged emotional distress [12]. A significant positive association between help-seeking and CST duration would also provide data on the construct validity of our duration measure as an indicator of trauma severity.

### 1.4. The National Sexual Health Survey (NSHS)

We searched published literature and data archives (last 30 years) for datasets with a representative sample of U.S. adults, and which included a direct assessment of CST duration along a time-based dimension. Furthermore, we needed a sample size large enough to provide sufficient power for assessing differences in relatively lower prevalence AAOs across sub-populations of CST survivors. We identified one study that fit these criteria: the National Sexual Health Survey (NSHS; 1995–96). The NSHS assessed the duration (i.e., months) of CST experiences with up to five separate perpetrators, and a wide range of AAOs across multiple life domains, among a large, probability-based sample of U.S. adults. As a result, we could delineate specific, time-based duration categories including those that have low prevalence (e.g., CST experiences of extremely long duration). This allowed us to identify and characterize chronic CST survivor sub-groups.

Historical studies, despite their age, are beneficial for examining theoretical relationships between variables [53,54]. The NSHS’s distinct advantages increased confidence in the data set as useful for providing tests of the long-term relationships hypothesized by traumatic stress theory. A potential challenge, however, is the limited generalizability of CST prevalence estimates to current populations. We will explore this concern by comparing CST prevalence in the NSHS to subsequent independent cross-sectional studies using probability-based samples and comparable CST definitions (see Results). The stability of CST prevalence over time would support the generalizability of NSHS estimates to more current cohorts of U.S. adults (see Discussion for other limitations).

### 1.5. Study Purpose

We examined the effects of CST duration on the occurrence of AAOs (physical and mental health, close relationships, and life achievement) and trauma-related help-seeking in a probability sample of U.S. adults. We hypothesize that individuals reporting longer duration CST will evidence a greater risk of cumulative AAOs and more engagement in help-seeking activities. Furthermore, we explored the generalizability of CST prevalence estimates from the NSHS by comparing them with estimates from recent probability samples of U.S. adults.

## 2. Materials and Methods

### 2.1. Sampling, Data Collection, and Procedures

The NSHS (1995–96) was a random digit-dial (RDD) survey (cooperation rate = 65%) with interviews (approx. 1 h) conducted in English and Spanish for U.S. adults [55]; detailed procedures are available online [56]. Participants in the current analyses include those aged 18–70 years (*N* = 6537; sample exclusions described in Table 1 notes; demographic characteristics reported in Table 2). Numerous studies have used RDD interview methods to assess CST, including the National Violence Against Women Survey [57], the National Alcohol Survey [24], and the Urban Men’s Health Study [25,37], and produced similar CST estimates. Telephone interviews have been found to generate high quality data on sensitive topics [58,59].

### 2.2. Measures

#### 2.2.1. Independent Variable: CST Duration

We operationalized severity in terms of total trauma duration. The direct, time-based CST duration measure in the NSHS allowed us to use a taxometric-based approach [60] to generate specific trauma duration categories. That is, we derived a categorical measure of CST duration from its continuous form, in order to identify priority sub-populations with the greatest long-term health risk [61,62].

CST history was assessed using a screener item: “Have you ever been forced or frightened into doing something sexually that you did not want to do?”. Each person responding “yes” to the screener was asked, “How many different people have forced or frightened you into doing something sexually that you did not want to do?”; a series of perpetrator-specific questions was asked for up to five perpetrators (e.g., respondent’s age at the time of the experience), beginning with the person who first assaulted the respondent. Participants responding “yes” and indicating at least one experience before age 18 were considered to have experienced CST (*n* = 587, 8.9%; No CST: *n* = 5950, 91.1%). This approach has been utilized in both clinical and research settings [25,37,40], demonstrating construct, predictive, and known groups validity.

The CST duration variable was derived from two perpetrator-specific questions: “Did this person do this to you more than once?” (if No and only one CST experience reported: CST duration = single event) and “Over how long a time period did it happen?” (<12 m., 12–23 m., ≥24 m.). Duration was computed by subtracting the participant’s age at their first CST encounter from their age at the final CST encounter and adding the duration of their final CST encounter (No CST = 0 years Duration). We first constructed a continuous CST duration measure (i.e., total trauma duration in whole years; Range = 1–17 years; M = 2.3 years, SD = 2.9 year), from which we identified cut points for the purposes of categorization by: (a) assessing differences between adjacent duration values (0, 1, 2, … ≥5 year; logistic regression models) with respect to all AAOs; and (b) collapsing adjacent duration values which were not significantly different with respect to any of the AAOs. Final categories included: no CST (*n* = 5950; 91.3%), single event (*n* = 236; 3.6%), intermediate duration (i.e., 1–3 years; *n* = 238; 3.7%), and extreme duration (i.e., ≥ 4 years; *n* = 94; 1.4%).

#### 2.2.2. Dependent Variables and Demographic Covariates

Detailed descriptions of all dependent and sociodemographic control variables are provided in Table 1.

### 2.3. Data Analysis

SPSS and Stata15 software were used for data management and analyses, respectively. Design and post-stratification weights were applied to demographic and prevalence estimates. All other analyses were performed on unweighted data. We used multiple imputation to adjust for missing data required for the assignment of individuals to a sexual orientation group. We examined CST duration in relation to each of the AAOs (logistic regression), the cumulative AAO measure (OLS regression), and reports of help-seeking (omnibus chi-square tests and logistic regression). Fit was examined using the Pearson test (*p* > 0.05), as recommended for logistic regression with large sample data (data available from first author) [67]. Logistic regression models with small sub-groups (n < 30) were analyzed using penalized likelihood estimation methods [68]. All regression models control for significant background variables (i.e., age, gender, race/ethnicity, and sexual orientation).

## 3. Results

### 3.1. CST Exposure and Duration Category Prevalence

Approximately 8.1% of participants aged 18–70 years [weighted data; 95% confidence interval (CI): 7.4, 8.9] reported experiencing CST prior to age 18. The highest prevalence was reported among women aged 18–29 years [20.8% (95% CI: 18.0, 24.1)], lesbian/bisexual women [45.5% (95% CI: 35.5, 56.6)], and gay/bisexual men [18.9% (95% CI: 11.3, 29.8)] (Table 3). Regarding CST duration, extreme duration was significantly less prevalent [1.4% (95% CI: 1.1, 1.7)] than either single event [3.1% (95% CI: 2.7, 3.6); Wald F_1, 6,517_ = 41.7, *p* < 0.001] or intermediate duration CST [3.4% (95% CI: 3.0, 3.9); Wald F_1, 6,517_ = 48.3, *p* < 0.001].

### 3.2. CST Duration: Cumulative AAOs in Adulthood

We examined the cumulative number of AAOs across three domains (physical and mental health and achievement) by totaling each AAO reported and computing mean values for comparison (Range = 0–9 AAOs; M: 1.7, SD: 1.4). Since not every respondent had been or was currently married/in a primary partnership, our cumulative total did not include the relationship indicators. In a sub-analysis, we compared mean cumulative AAO values by duration group for an indicator based on only the subset of individuals who had ever been married, and which included the relationship indicators, and found no difference in the patterns observed (data available from first author).

Participants with CST exposure reported significantly more AAOs than those without CST (CST: M = 2.4, SD = 0.07; No CST: M = 1.6, SE = 0.02; *p* ≤ 0.001). We compared the mean number of AAOs across duration categories (OLS regression) controlling for demographic differences. The accumulation of AAOs increased significantly with longer CST duration. That is, the mean number of AAOs increased with longer duration, with significant differences between all duration categories (single event CST: M = 2.1, SE = 0.1; intermediate duration: M = 2.4, SE = 0.1; extreme duration: M = 3.0, SE = 0.2; all *p* ≤ 0.05).

To contextualize CST related sexual health problems, we examined associations between sexual functioning and other AAOs (emotional and physical health problems, income challenges, and educational attainment (adjusted logistic regression; relationship AAOs and adult sexual trauma not included due to sample exclusions, see above and Table 1 note). For participants with CST histories, the odds of sexual dissatisfaction and sexual dysfunction were higher among those with emotional [OR = 3.0 (95% CI = 2.0, 4.4) and OR = 3.0 (95% CI = 1.8, 4.9), respectively] and physical health problems [OR = 1.9 (95% CI = 1.3, 2.9) and OR = 2.2 (95% CI = 1.3, 3.6)], fair/poor overall health [OR = 2.8 (95% CI = 1.6, 4.7) and OR = 3.2 (95% CI = 1.8, 5.8)], and STI history [OR = 1.8 (95% CI = 1.2, 2.7) and OR = 2.0 (95% CI = 1.2, 3.4)]. Sexual dysfunction was also associated with poverty status [OR = 1.6 (95% CI = 1.0, 2.6)], and showed a significant trend in relation to lower education [OR = 1.7 (95% CI = 0.9, 3.2)].

### 3.3. Does AAO Prevalence Increase with Longer CST Duration?

All models, as noted previously, control for significant background characteristics (see Table 4 note). Relative to participants reporting no CST, all three duration categories had significantly larger relationships in 12/15 AAO models; single event CST and no-CST groups were not significantly different in three models (male sexual dysfunction, lower education, and poverty status; see Table 4). We compared CST duration categories (logistic regression; first, we examined CST duration as a continuous variable and found significant effects for all 15 AAOs; see Table 4) and found that for a large majority of models (13/15), there was a general increase in AAOs with longer CST duration. In only two models (lifetime STIs and adult sexual trauma) did all three duration categories have similar relationships to the outcome. In brief, longer CST duration was associated with an increased likelihood of reporting recent emotional and physical health problems, sexual dissatisfaction and dysfunction (especially for women), fair/poor overall health, current divorce/separation, a history of extra-marital sex, current relationship challenges, lower educational attainment, income below poverty level, and a history of incarceration.

### 3.4. Cumulative Help-Seeking Related to CST

Among participants reporting CST, approximately 38% sought some type of CST-related help (i.e., self-, informal-, and/or formal-help; see Table 5). Help-seeking overall increased significantly with longer CST duration [single event: 27%; intermediate duration: 42%; extreme duration: 59%; Omnibus χ^2^ (2) = 31.9, *p* ≤ 0.001; all between-category differences significant at *p* ≤ 0.05]. Participants sought more help from formal (31%) than informal sources (19%; e.g., friends or family). Reports of formal help-seeking increased significantly with longer CST duration [single event: 20%; intermediate: 34%; extreme: 54%; Omnibus χ^2^ (2) = 37.8, *p* ≤ 0.001; all between-category differences significant at *p* < 0.01]. In terms of formal help sources, reports of seeking help from mental health providers increased significantly with longer CST duration (all between-category differences significant at *p* < 0.01), and help-seeking from spiritual leaders was more common among the extreme duration category than the intermediate and single event categories (*p* ≤ 0.001; see Table 5). Informal help-seeking also showed an increasing trend with longer duration [single event: 12%; intermediate: 23%; extreme: 26%; Omnibus χ^2^ (2) = 11.7, *p* = 0.003]. Reports of help-seeking were not related to the cumulative number of AAOs [Sought help: M = 2.4 AAOs, SD = 1.6; No help: M = 2.5 AAOs, SD = 1.7; t(571) = *−*1.14, *p* = 0.25].

### 3.5. CST Prevalence Trends

Our comparison of the NSHS with two subsequent studies suggests that CST prevalence has not changed dramatically over recent decades among U.S. adults and specific high risk sub-populations (see Table 6). For instance, among adult women (age ≥ 18 years.), CST prevalence was similar (i.e., overlapping confidence intervals) in the NSHS [1996; 13.7% (95% CI: 12.4, 15.0)] and the NAS [2005; 12.3% (95% CI: 11.2, 13.4)]; both estimates were approximately 5–6% lower than CST prevalence reported in the NESARC-III (2013; 18.2%, 95% CI: 17.6, 18.8). Among men-who-have-sex-with-men (MSM), CST prevalence was similar between the NSHS [1996; 18.9% (95% CI: 11.3, 29.8)] and the UMHS-III [2003; 22.0% (95% CI: 20.6, 23.4)].

## 4. Discussion

### 4.1. Overview

Our study illustrates that CST experiences of chronic duration have more robust relationships to AAOs relative to single-exposure traumas. Trauma-related help-seeking also increased with longer CST duration (providing construct and concurrent validity support for our CST duration measure), but large proportions of those with intermediate or extreme duration trauma have never sought help. These groups would benefit from efforts to improve help-seeking, trauma screening, and dissemination of trauma-informed programs. This work advances our understanding of trauma-related screening efforts by providing support for the use of multi-level rather than aggregate exposure assessment tools (see below). Lastly, we address an important research goal by characterizing CST duration into discrete hierarchical categories that aid identification of populations at high priority for intervention.

### 4.2. Chronic CST: Long-Term Health & Sexual Health Outcomes

Our results support trauma theory with regards to the importance of the temporal components of stressors in determining stress outcomes, the importance of chronic trauma in producing more severe and long-term AAOs, and the observation that single event traumas, although stressful, have less impact on long-term AAOs. These findings suggest that traumas of longer duration facilitate the development of maladaptive pathways influencing a broad spectrum of AAOs [12]. Chronic trauma experiences are believed to produce repeated learning trials—associated with increasingly complex stimuli and anticipatory fears between incidents—which act to strengthen and generalize traumatic stress responses to environmental stimuli that, in turn, sustain long-term adverse outcomes [12,70]. Although some research has explored pathways between CST and AAOs [37,44,71,72], further research is needed to understand when to intervene on childhood and adolescent developmental processes that are negatively affected by trauma. Furthermore, it is important to gain an understanding of conditions that sustain these processes over adulthood and to identify factors that contribute to healing and resilience.

From a sexual health perspective, these underlying considerations may characterize the difficulties CST clients with more severe trauma histories have in resolving sexual difficulties. The challenge is that the trauma may not only have a direct impact on one’s sexual relationships, but a more general effect on mental health and other relationship stressors that can adversely impact the quality of one’s sexual life. Findings from the current study indicated relationships between sexual functioning, physical and emotional health, and achievement AAOs among CST survivors. That is, the wide-reaching effects of CST on well-being may themselves compound the direct impact of the trauma on sexual functioning (e.g., through the impact of physiological, affective, and cognitive response processes on sexual arousal; [31,32]). This aligns with recent research suggesting that health and relationship functioning contribute to associations between CST and sexual difficulties in adulthood [28,33]. In general, interrelationships among life stressors, relationship quality, and psychological health may facilitate sexual difficulties [29,73,74]. It is possible that CST further complicates these relationships through adverse effects on stress response processes and coping strategies [30,75,76]. Further research is needed to understand the mechanisms of CST’s impact on adult sexual health, which addresses methodological and sampling inconsistencies that have challenged this area of work [28,77].

### 4.3. CST & Help-Seeking

The relationship between help-seeking and CST duration provides a measure of construct validity for our CST severity indicator. That is, help-seeking increases with longer duration CST. Our findings are consistent with theoretical and empirical work indicating that help-seeking behavior is strongly motivated by problem-related distress [52,78–80]. An important clinical goal is to develop programs to increase help-seeking among individuals with CST histories, particularly those at highest risk for AAOs. We found a large proportion (62%) of people with CST were unlikely to seek help related to those traumatic experiences (see Limitations), and a large subsegment of CST survivors did not seek help from clinical professionals. Programs designed to intervene in this subpopulation with strategies that increase help-seeking behavior, particularly help-seeking from mental and physical health providers [48,81], have either not been developed or widely implemented.

### 4.4. Measurement Theory: Research and Screening

The NSHS allowed us to generate and compare categories of chronic CST survivors in terms of AAO risk. Prior work assessing CST duration among large representative samples is scant and has lacked measures that facilitate a time-based delineation of chronic CST experiences. Broadly, past studies have operationalized duration as dichotomies (any vs. none; e.g., see [82], within blended measures that obscure duration’s effects (e.g., a CST severity score that includes duration along with other severity indicators such as the victim’s age or relationship to the perpetrator) [83], or as subjective frequencies (e.g., the NESARC-III used a categorical measure of subjective CST frequency [i.e., never, almost never, sometimes, fairly often, very often]) [69]. Subjective assessments, however, conflate the actual duration of trauma exposure with survivors’ perceptions of time. This impedes the discrimination of specific trauma duration categories and the identification of chronic CST survivor populations with greater long-term health risk. Categorization is beneficial for informing treatment decisions and, to some extent, prevention goals, which are often categorical (e.g., assessing intervention effectiveness by the proportion of cases above and below a clinical cutoff score) [61]. Future work might aim to continue refining time-based duration categories and examining their association with physical and social-psychological outcomes across development.

A consideration of chronic trauma stands in contrast to “single exposure” or aggregate assessments for establishing trauma risk. For instance, there is a growing recognition that the Adverse Childhood Experiences (ACEs) instrument, and similar additive scales of trauma experience, may be limited in their utility [26,84–86]. Findings from a recent longitudinal study underscore the importance of this perspective, and illustrate the limitations of the ACEs measure in predicting long-term adverse outcomes [87]. In particular, such global measures fail to discriminate CST sub-populations, and obscure elements of individual trauma experiences (e.g., chronicity) that influence the development and accumulation of long-term adverse outcomes. Global trauma measures may be useful as an initial screening tool, to be followed by screening positive cases with more intensive methods that assess trauma severity. These second-level screening tools will aid in identifying individuals with greater levels of trauma severity-related health outcomes.

### 4.5. NSHS Generalizability

Our findings also suggest that CST prevalence estimates have not changed dramatically over recent decades. The small variation across the independent samples we examined may reflect methodological differences [88–90] or changes in trauma disclosure over time [91,92]. The consistency of CST prevalence over 30 years supports the generalizability of NSHS estimates. Moreover, the persistence of CST over time and the size of survivor subpopulations (see Population Impact) underscore (a) the enormous impact of CST on the overall health of the nation, (b) the importance of trauma-related help-seeking research, and (c) the importance of sustained multilevel screening for trauma-related experiences.

### 4.6. Population Impact

Approximately 8% of NSHS participants reported a CST history; applying NSHS prevalence estimates to U.S. census data for 2000 (proximal to the NSHS survey) [93] illuminates the public health burden of CST on the U.S. population. Approximately 15 million U.S. adults (18–69 years) in 2000 had a history of CST (single event = ~5.6 million, intermediate duration = ~6.2 million, extreme duration = ~2.6 million). Extrapolating to 2018 (using 2018 Current Population Survey) [94], we would estimate a growth of approximately 1 million in the overall CST survivor population (i.e., overall CST = approx. 16 million adults, 18–64 years; single event = ~6.1 million, intermediate duration = ~6.7 million, extreme duration = ~2.8 million). It is possible that the CST survivor population is even larger considering that NSHS confidence intervals suggest CST prevalence may be 3–5% higher, and that CST may be underreported [90,91].

### 4.7. Strengths and Limitations

Large historical datasets such as the NSHS offer useful information about theoretically important relationships between variables. Historical data sets were used frequently at the beginning of the HIV epidemic to provide data on the distribution of sexual orientation groups in the population and to examine important developmental relationships between CST and sexual health outcomes (e.g., [95,96]). In a reproductive health context, Hyland et al. [97] examined lifetime second-hand tobacco exposure in relation to pregnancy outcomes, using a 20-year-old dataset that had specific benefits over subsequent studies (e.g., more refined measures that allowed for lifetime tobacco exposure assessment). In general, small sample sizes have often limited CST research [19,44,83]. The NSHS dataset is valuable because of its large sample size, detailed CST assessments that used a seldom-employed enumeration method, and assessment of a wide range of AAOs in a single investigation.

NSHS prevalence estimates should be generalized with caution, although we provide data suggesting that CST estimates may be relatively stable (see Table 6). Furthermore, although our help-seeking results may not be generalizable to current cohorts, recent research suggests there is also little reason to believe that CST-related help-seeking levels have changed significantly [46,47,98]. Although this may be true for professional help-seeking, other technologically based help sources (i.e., the internet) were not as prevalent when the NSHS was conducted. With regards to help-seeking, our study was unable to examine the developmental pathways linking CST, adverse outcomes, and help-seeking.

The current study also has all the accompanying limitations associated with cross-sectional studies and self-report measures. The sequential time periods involved (i.e., child/adolescent CST, adult AAOs) allow for some presumption of causality, but memory lapses, avoidance/denial coping, and other cognitive processes may inhibit event recall. We also did not assess other types of traumas that co-occur with CST or in addition to CST events. However, prior work has found CST to have more robust [24,43,71,99] or unique relationships to AAOs [15,25,100] compared to other types of traumas (e.g., physical abuse, psychological abuse, and neglect). Furthermore, there may be physical and/or psychological elements of CST experiences which contribute to the observed effects. As such, insight into the effects of CST experiences may also provide insight into the effects of physical and/or psychological trauma on adult outcomes. Future work should continue to explore trauma chronicity in relation to long-term health and help-seeking trajectories, for individuals with CST both with and without other childhood traumatic experiences.

## 5. Conclusions

Our study builds on prior work demonstrating the array of long-term health risks associated with CST [1,3,4] and highlights the importance of examining AAOs that span multiple life domains. We examined CST in relation to AAOs across four major life domains, which allowed us to identify survivors experiencing the highest degree of impairment. Our findings suggest that adverse sexual health outcomes among CST survivors may be linked with both the trauma itself and the impact on related aspects of well-being, particularly for those with chronic trauma histories. Furthermore, our work demonstrates a need for interventions that facilitate CST-related help-seeking over the life course, and particularly for individuals with chronic CST histories. The current findings suggest that trauma duration has value as a tool for delineating CST survivors with the greatest long-term health risk, supporting the public health goal of identifying high priority subpopulations.

## Figures and Tables

**Table 1.** Measures and final variable coding for dependent and sociodemographic control variables (National Sexual Health Survey, 1996) ^†^.

Adult Adverse Outcomes (AAOs)
Mental Health Outcomes
Mental Health Problems [63] “In the past 4 weeks have you had any emotional problems?” [1 = Yes (n = 617, 9.5%), 0 = No (n = 5912, 90.5%)]
**Adult Adverse Outcomes (AAOs)**
Adult Sexual Trauma Screener: “Have you ever been forced or frightened by someone into doing something sexually that you did not want to do?”; Participants responding “yes” and who were exposed to at least one experience of sexual trauma at age 18 or older. [1 = Yes (*n* = 606, 8.7%), 0 = No (*n* = 6389, 91.3%)]
Low Sexual Satisfaction ^a^ “In the last 12 months, has there been something either physical or emotional that has made it difficult for you to have a satisfying sexual relationship?” [Gender-specific variables; Women: 1 = Yes (*n* = 598, 18.1%), 0 = No (*n* = 2704, 81.9%); Men: 1 = Yes (*n* = 422, 13.4%), 0 = No (*n* = 2730, 86.6%)]
Sexual Dysfunction (Females) ^a^ [64] Composite index of orgasmic and arousal difficulties; “Over the last 12 months how often have you had difficulty having an orgasm?” (1 = Never to 4 = All the time); “When you have sex is it easy or difficult for you to get sexually aroused?” (1 = Very easy to 4 = Very difficult) [1 = Moderate/severe dysfunction (scores of 3–4 on orgasm difficulty and/or arousal; *n* = 249, 7.6%); 0 = Low/no dysfunction (*n* = 3035, 92.4%)]
Sexual Dysfunction (Males) ^a^ [64] Composite index of erectile, orgasmic, and arousal difficulties; “Over the last 12 months how often have you had difficulty having an orgasm?” (1 = Never to 4 = All the time); “Over the last 12 months, how often have you had difficulty keeping an erection?” (1 = Never to 4 = All the time); “When you have sex is it easy or difficult for you to get sexually aroused?” (1 = Very easy to 4 = Very difficult) [1 = Moderate/severe dysfunction (scores of 3–4 on orgasm, erectile, and/or arousal difficulty; *n* = 127, 4.0%); 0 = Low/no dysfunction (*n* = 3014, 96.0%)]
Physical Health Outcomes
Global Health ^b^ [63] “How would you rate your overall health?” [1 = Fair/poor (*n* = 604, 9.2%); 0 = Good/very good/excellent (*n* = 5932, 90.8%)]
Recent Health Problems [63] “In the past 4 weeks have you had any physical health problems?” [1 = Yes (*n* = 964, 14.8%), 0 = No (*n* = 5571, 85.2%)]
Lifetime STI diagnosis “Have you ever had a doctor or nurse tell you that you have a venereal disease?” [1 = Yes (*n* = 610, 9.3%), 0 = No (*n* = 5919, 90.7%)]
Close Relationship Outcomes
Divorce/Separation ^c^ “Currently are you...?” (1 = Legally married to 5 = Never married) [1 = Divorced/separated (*n* = 1001, 20.4%), 0 = Married/widowed (*n* = 3899, 79.6%)]
Extramarital sex (EMS) ^c^ “At any time during your (1st-5th marriage) did you have sex with someone other than your husband/wife?” [1 = Any EMS (*n* = 963, 19.8%), 0 = No EMS (*n* = 3907, 80.2%)]
Relationship Closeness ^d^ [65] 8 item scale assessing relationship closeness (e.g., “We have a regular time for getting together”; “We each know what we expect of each other”; 1 = Agree a lot to 4 = Disagree a lot); mean item scores were computed for each participant, with higher scores = greater closeness (Cronbach’s α = 0.69). Individuals with scores 1 or more standard deviations below the mean were operationalized as low scores (M = 27.0, SD = 3.8) and participants were dichotomized into low and high closeness groups. [1 = Low closeness (*n* = 699, 15.3%), 0 = High closeness (*n* = 3874, 84.7%)]
Achievement Outcomes
Lower Education “What was the highest grade/year of school you completed?” [1 = < College degree (*n* = 1973, 30.2%), 0 = College degree or higher (*n* = 4562, 69.8%)]
Poverty Status [66] “In general, people with larger incomes can more easily get medical care. Tell me when I get to the category that best describes your household income before taxes for all of 1994. Please include the income of everyone in your household who contributed to your household income.” (1 = *≤* $10,000 to 6 = > $80,000) [Income below poverty level estimated based on census poverty threshold for 1994 (~$16,000); 1 = *≤* $20,000 (*n* = 1606, 25.8%), 0 = > $20,000 (*n* = 4630, 74.2%)]
Incarceration “Have you ever been in jail for more than 24 h anytime in the last 15 years?” [1 = Yes (*n* = 341, 5.2%), 0 = No (*n* = 6188, 94.8%)]
**Adult Adverse Outcomes (AAOs)**
Cumulative AAOs ^e^ Mean score variable reflecting cumulative AAOs across physical and mental health and achievement domains (Range = 0–9 AAOs; M: 1.7, SD: 1.4).
Lifetime Help-Seeking: CST-Related
Sought Help Participants reporting forced/frightened sex were asked: “These are very personal experiences that some people keep to themselves. Other people seek some kind of help. By help we mean advice, information, or counseling. Did you seek help?” [Among participants with CST: 1 = Yes (*n* = 222, 37.9%), 0 = No (*n* = 364, 62.1%)]
Help Sources “Who did you seek help from?” (participants instructed to select all that apply: police, teacher, clergy/spiritual leader, counselor/therapist/psychiatrist, medical doctor/nurse, magazines/newspaper/TV/radio, call-in radio, parent, spouse, other relative, friend) [2 variables were created to reflect types of help sought: any formal help and any informal help. Any formal help included police, teacher, clergy/spiritual leader, mental health providers, and medical providers (1 = Sought any formal help, *n* = 179, 30.5%; 0 = Did not seek formal help, *n* = 407, 69.5%). Informal help included parent, spouse, other relative, and friend (1 = Sought any informal help, *n* = 107, 18.8%; 0 = Did not seek informal help, *n* = 478, 81.4%). Participants who reported no help-seeking were included in the “did not seek help” group for both variables.]
**Sociodemographic Control Variables**
Age: Continuous, years (M: 39.8, SD: 0.17)
Gender: Dichotomous, 1 = Male, 0 = Female
Race/ethnicity: Dichotomous item derived from two separate items assessing racial (“Which would you say best describes your racial background?”) and ethnic identity (“Are you of Spanish, Hispanic, or Latino origin?”).
Sexual orientation: Derived from items assessing participant gender and the gender identity of sexual partners in since age 18.

† *Note:* For all variables, we report the number and proportion of participants among the total sample (*n* = 6537). CST experiences where the perpetrator was reported to be a spouse or <10 years old (*n* = 8) were excluded. Analyses of adult sexual trauma were performed on an expanded sample (*n* = 7028) which included participants who reported adult sexual trauma without CST (*n* = 471; excluded from all other analyses). The NSHS included a rigorous survey development process; all measures were extensively pre-tested using both qualitative and quantitative techniques [56].

a Sexual health measures were developed by an advisory panel of clinical sex therapists and researchers for the NSHS. The term “venereal disease” was determined from extensive pre-testing to be the most recognizable way of describing sexually transmitted infections at the time the survey was conducted. We aggregated sexual dysfunction measures because specific sexual dysfunctions have a relatively low prevalence.

b A global health assessment is useful because CST survivors often evidence a wide range of health problems in adulthood [2–4].

c Assessed among participants reporting at least one marriage.

d Assessed among participants reporting a current relationship with a primary partner.

e In computing this measure, we (a) created non-gender-specific measures for sexual dysfunction and sexual dissatisfaction; and (b) excluded outcomes that significantly reduced the sample frame or posed methodological problems (i.e., those requiring individuals to have been married/in a primary relationship, and adult sexual victimization.

**Table 2.** Demographic characteristics: Total sample and CST duration sub-groups (National Sexual Health Survey, 1996) ^†^.

Demographic Characteristics (Reference Group)	Total Sample (*N* = 6537)	Single Event CST (*N* = 236)	Intermediate Duration (*N* = 238)	Extreme Duration (*N* = 94)
	% (*N*)	% (*N*)	% (*N*)	% (*N*)
Gender				
Female	48 (3169)	77 (189)	81 (199)	87 (87)
Male (ref)	52 (3368)	23 (47)	19 (39)	13 (7)
Age group				
18–29 years	27 (1674)	37 (86)	37 (81)	42 (35)
30–39 years	26 (1824)	34 (83)	32 (80)	39 (38)
40–49 years	21 (1486)	17 (42)	19 (52)	14 (15)
*≥*50 years (ref)	26 (1553)	11 (25)	12 (25)	6 (6)
Race/ethnicity				
White (ref)	73 (4996)	80 (191)	75 (184)	75 (71)
Black	12 (605)	10 (21)	15 (26)	6 (6)
Hispanic/Latino	10 (605)	7 (16)	7 (16)	10 (11)
Other	6 (329)	3 (8)	3 (8)	9 (6)
Sexual orientation				
Heterosexual (ref)	98 (6156)	94 (218)	89 (207)	86 (80)
Gay/lesbian/bisexual	2 (218)	6 (15)	11 (29)	14 (13)
Education				
≤HS diploma/GED	43 (2815)	49 (91)	59 (114)	65 (53)
Some college	27 (1747)	31 (82)	28 (79)	22 (23)
*≥*College degree (ref)	30 (1973)	20 (63)	13 (45)	13 (18)

† Note: Design and post-stratification weights applied to achieve sample characteristics and demographic distributions.

**Table 3.** CST prevalence: Total sample and demographic sub-groups (National Sexual Health Survey, 1996) ^†^.

Demographic Characteristics (Reference Group)	CST Prevalence		
	Total Sample (*N* = 6537)	Women (*n* = 3368)	Men (*n* = 3169)
	% [95% CI]	% [95% CI]	% [95% CI]
Gender			
Female	13.7 [12.4, 15.0] ***	–	–
Male (ref)	3.0 [2.4, 3.8]	–	–
Age group			
18–29 years	11.0 [9.5, 12.8] ***	20.8 [18.0, 24.1] ***	2.3 [1.4, 3.7]
30–39 years	10.7 [9.3, 12.4] ***	17.0 [14.6, 19.8] ***	5.1 [3.6, 7.1] **
40–49 years	6.9 [5.7, 8.4] ***	11.3 [9.1, 14.0] ***	2.8 [1.8, 4.4]
*≥*50 years (ref)	3.5 [2.6, 4.6]	5.0 [3.6, 6.9]	1.9 [1.0, 3.6]
Race/ethnicity			
White (ref)	8.6 [7.8, 9.5]	14.7 [13.2, 16.3]	3.2 [2.5, 4.1]
Black	7.7 [5.7, 10.4]	11.6 [8.4, 15.9]	2.1 [0.9, 5.0]
Hispanic/Latino	6.1 [4.4, 8.2] *	9.8 [6.9, 13.7] **	2.9 [1.5, 5.5]
Other	6.5 [4.2, 9.9]	11.4 [7.1, 17.8]	3.1 [1.2, 7.8]
Sexual orientation			
Heterosexual (ref)	7.6 [7.0, 8.4]	13.1 [11.9, 14.5]	2.6 [2.0, 3.3]
Gay/lesbian/bisexual	31.1 [24.4, 38.7] ***	45.9 [35.5, 56.6] ***	18.9 [11.3, 29.8] **
Education			
*≤*HS diploma/GED	8.5 [7.4, 9.6] ***	1 4.0 [12.2, 16.0] *	3.3 [2.4, 4.5] **
Some college	9.8 [8.3, 11.4] ***	15.0 [12.8, 17.6] *	4.4 [3.0, 6.3] **
*≥*College degree (ref)	5.7 [4.7, 6.9]	11.2 [9.2, 13.5]	1.3 [0.7, 2.2]

† Note: Design and post-stratification weights applied to achieve CST prevalence estimates. Adjusted Wald F tests compared CST prevalence estimates across demographic groups (weighted sample, reference groups indicated). Analyses of demographic differences between CST duration groups found no significant differences (weighted sample; data available from first author).

* *p ≤* 0.05,

** *p ≤* 0.01,

*** *p ≤* 0.001.

**Table 4.** Adverse outcomes by CST duration (National Sexual Health Survey, 1996) ^†^.

Chronic CST Duration								
Adult Adverse Outcomes	No CST (*N* = 5950)	CST Duration: Continuous	Single Event CST (*N* = 236)		Intermediate Duration (*N* = 238)		Extreme Duration (*N* = 94)	
	*%*	aOR [95% CI]	%	aOR [95% CI]	%	aOR [95% CI]	%	aOR [95% CI]
Mental Health								
Mental Health Problems (Yes)	8	1.2 [1.1, 1.3] ***	17	1.9 [1.3, 2.7] ***	23	2.7 [2.0, 3.7] ***	33	4.2 [2.7, 6.6] ***
Adult Sexual Trauma (Yes)	7	1.5 [1.4, 1.6] ***	22	2.3 [1.7, 3.3] ***	24	2.4 [1.7, 3.3] ***	26	2.1 [1.3, 3.5] **
Low Sexual Satisfaction (F)	16	1.2 [1.2, 1.3] ***	27	2.0 [1.4, 2.8] ***	30	2.3 [1.6, 3.1] ***	47	4.6 [3.0, 7.2] ***
Low Sexual Satisfaction (M)	13	1.2 [1.0, 1.4] *	23	2.4 [1.2, 4.7] *	26	2.8 [1.3, 5.7] **	33	4.3 [0.9, 20.7] ˆ
SxDys (F) (Moderate/Severe)	6	1.2 [1.1, 1.3] ***	13	2.3 [1.4, 3.5] ***	16	2.8 [1.9, 4.3] ***	21	4.0 [2.3, 6.9] ***
SxDys (M) (Moderate/Severe)	4	1.3 [1.1, 1.5] **	4	1.5 [0.4, 6.0]	13	4.7 [1.7, 13.0] **	17	8.4 [1.0, 69.8] *
Physical Health								
General Health (Fair/poor)	9	1.2 [1.1, 1.2] ***	10	1.8 [1.2, 2.8] **	10	1.6 [1.0, 2.5] *	18	3.6 [2.1, 6.2] ***
Health Problems (Yes)	14	1.1 [1.1, 1.2] ***	17	1.4 [1.0, 2.0] ˆ	22	1.8 [1.3, 2.5] ***	31	2.8 [1.8, 4.4] ***
STI (Ever)	8	1.2 [1.1, 1.3] ***	20	3.2 [2.3, 4.5] ***	22	3.5 [2.5, 4.9] ***	21	3.3 [2.0, 5.6] ***
Close Relationships								
Divorced/Separated (Current)	19	1.1 [1.1, 1.2] ***	30	1.8 [1.3, 2.7] ***	29	1.7 [1.2, 2.4] **	40	2.7 [1.7, 4.4] ***
EMS (Ever)	19	1.2 [1.1, 1.3] ***	29	2.6 [1.8, 3.8] ***	33	3.2 [2.2, 4.6] ***	33	3.3 [1.9, 5.6] ***
Closeness (Low)	14	1.1 [1.0, 1.2] **	22	1.5 [1.1, 2.2] *	23	1.6 [1.1, 2.4] **	28	2.1 [1.2, 3.5] **
Achievement								
Ed. < College degree	69	1.1 [1.1, 1.2] ***	73	1.2 [0.9, 1.7]	81	2.0 [1.4, 2.8] ***	81	1.9 [1.1, 3.2] *
Poverty status (Current)	25	1.1 [1.1, 1.2] ***	26	1.0 [0.7, 1.3]	34	1.4 [1.0, 1.8] *	46	2.2 [1.5, 3.4] ***
Incarceration (Past 15 years.)	5	1.2 [1.1, 1.3] ***	7	2.8 [1.6, 4.9] ***	6	2.6 [1.4, 4.7] ***	9	5.2 [2.3, 11.7] ***

† Note: CST = childhood sexual trauma; Intermediate duration = 1–3 years; Extreme duration = ≥ 4 years; % = proportion of CST exposure or duration sub-group reporting outcome; aOR = adjusted odds ratio, logistic regression (reference group: no CST); 95% CI = 95% confidence interval; Mental health problems = past 4 weeks; SxDys = sexual dysfunction in females (F) and males (M); Health problems = past 4 weeks; STI = sexually transmitted infection, ever diagnosed; Divorced/Separated = current marital status; EMS = extra-marital sexual behavior; Closeness = low closeness with primary partner; Ed. < college degree = educational completion less than college degree; Income < poverty = income below approximate 1994 federal poverty level ($20,000 or less). All models adjusted for statistically significant demographic correlates (age, gender, race/ethnicity, and sexual orientation). Male sexual satisfaction and sexual dysfunction models used penalized likelihood estimation procedures to adjust for low numbers of men reporting CST and sexual functioning outcomes. Continuous CST duration variable was transformed to a z-scored variable for inclusion in logistic regression models.

* *p ≤* 0.05,

** *p ≤* 0.01,

*** *p ≤* 0.001,

ˆ *p ≤* 0.10.

**Table 5.** Lifetime CST-related help-seeking by CST duration (National Sexual Health Survey, 1996).

Help Sources	Any CST (N = 587)	CST Duration			Omnibus Chi-Square ^1^	Post-Hoc Comparisons ^2^
		Single Event (N = 236)	Intermediate (N = 238)	Extreme (N = 94)		
	% (N)	% (N)	% (N)	% (N)	*p*-Value	
Overall Help-Seeking	37.9 (222)	26.7 (63)	42.0 (100)	59.1 (55)	≤0.001	Ext > Int > SE
Help Sources						
Self ^3^	2.4 (14)	1.3 (3)	1.7 (4)	7.5 (7)	0.008	Ext > Int/SE
Informal help ^4^	18.6 (109)	12.3 (29)	22.7 (54)	25.5 (24)	0.003	Ext/Int > SE
Friend	9.7 (57)	6.8 (16)	10.1 (24)	17.2 (16)	0.021	Ext > SE
Parents	7.0 (41)	6.4 (15)	8.8 (21)	5.4 (5)	0.492	—
Other relative	4.3 (25)	2.1 (5)	5.9 (14)	5.4 (5)	0.096	—
Spouse	3.2 (19)	0.9 (2)	5.0 (12)	5.4 (5)	0.009	Ext/Int > SE
Formal help ^5^	30.6 (179)	19.5 (46)	33.6 (80)	53.8 (50)	*≤*0.001	Ext > Int > SE
Counselor/therapist	26.1 (153)	15.3 (36)	29.8 (71)	46.2 (43)	*≤*0.001	Ext > Int > SE
Clergy/spiritual leader	4.8 (28)	2.5 (6)	3.8 (9)	14.0 (13)	*≤*0.001	Ext > Int/SE
Doctor/nurse	3.2 (19)	2.1 (5)	4.6 (11)	3.2 (3)	0.311	—
Police	2.9 (17)	2.5 (6)	2.5 (6)	5.4 (5)	0.341	—
Teacher	0.3 (2)	0.9 (2)	0 (0)	0 (0)	0.474	—

1 Fisher’s exact chi-square test used for all omnibus tests of CST duration by help sources; post-hoc comparisons conducted if omnibus *p*-value ≤ 0.05;

2 Logistic regression examined post-hoc comparisons of help-seeking across duration groups, adjusted for age, gender, race/ethnicity, and sexual orientation; significant if *p* ≤ 0.05; SE = single event, Int = Intermediate duration (1–3 years), Ext = Extreme duration (≥4 years), All = differences across all duration categories significant at *p* ≤ 0.05;

3 Self = magazine, newspaper, TV, & radio sources;

4 Informal = parents, other relative, friend, or spouse;

5 Formal = doctor/nurse, counselor/therapist, police, teacher, or clergy/spiritual leaders.

**Table 6.** CST prevalence trends among U.S. adults and high-risk sub-populations (1995–2013) ^†^.

	NSHS (1995–96)		UMHS III (2002–2003)		NAS (2005)		NESARC-III (2012–2013)	

	n/N	Prevalence % [95% C.I.]	n/N	Prevalence % [95% C.I.]	n/N	Prevalence % [95% C.I.]	n/N	Prevalence % [95% C.I.]
Women ≥ 18 years	491/3368	13.7 [12.4, 15.0]	–	–	433/3601	12.3 [11.2, 13.4]	3296/18,111	18.2 [17.6, 18.8]
Men ≥ 18 years	96/3169	3.0 [2.4, 3.8]	–	–	–	–	992/16,000	6.2 [5.8, 6.6]
Women 18–55 years	471/2811	16.0 [14.6, 17.6]	–	–	343/2489	13.8 [12.4, 15.2]	–	–
MSM *≥* 18 years	17/112	18.9 [11.3, 29.8]	193/879	22.0 [20.6, 23.4]	–	–	–	–

† *Note:* NSHS = National Sexual Health Survey; UMHS III = Urban Men’s Health Study III [37]; NAS = National Alcohol Survey [24]; NESARC-III = National Epidemiologic Studies of Alcohol Related Conditions-III [69]; n / N = number of sub-sample reporting CST / total sub-sample size; MSM = men-who-have-sex-with-men. CST definitions included unwanted sexual experiences before age 18 which involved force or fear (NSHS, UMHS, and NAS) or sexual contact before age 18 that was unwanted or which the respondent was too young to understand (NESARC-III). Unreported sample sizes, prevalence estimates, and/or confidence intervals were calculated by the first author using reported data and may be subject to inaccuracies. Upper age limits varied across studies (i.e., NSHS: 70 years; UMHS: 90 years; NESARC, NAS: unknown), so estimates may not be directly comparable. NSHS estimates are further corroborated by data from an independent sample of MSM in the UMHS I (1995–96): CST prevalence = 20.6% (95% CI: 18.8, 22.5) [17].

## Data Availability

The data that support the findings of this study are available in Sociometrics at https://www.socio.com/products/aids-petra-0204 (accessed on 1 June 2022).
